# Cytokeratin Expression Pattern in Human Endometrial Carcinomas and Lymph Nodes Micrometastasis: a Mini-review

**DOI:** 10.7150/jca.70550

**Published:** 2022-03-14

**Authors:** Danuta Vasilevska, Vilius Rudaitis, Aneta Adamiak-Godlewska, Anna Semczuk-Sikora, Dorota Lewkowicz, Dominika Vasilevska, Andrzej Semczuk

**Affiliations:** 1 Vilnius University Hospital “Santaros Clinics”, Department of Gynecology, Vilnius, Lithuania; 2 Faculty of Medicine, Vilnius University, Vilnius, Lithuania; 3 IIND Department of Gynecology, Lublin Medical University, Lublin, Poland; 4 Department of Pathology of Pregnancy, Lublin Medical University, Lublin, Poland; 5 Department of Clinical Pathology, Lublin Medical University, Lublin, Poland

**Keywords:** Cytokeratin, Endometrial Carcinomas, Lymph Node, Metastasis, OSNA.

## Abstract

Cytokeratins (CKs) are the largest subgroup of intermediate filament proteins, preferentially expressed in epithelial tissues. CKs play a critical role in determining epithelial structural integrity under stressful conditions in addition to their various fundamental functions in cellular proliferation, apoptosis, migration, adherence and molecular signaling. Immunohistochemical CKs staining could be evaluated with a proper comprehension of their task limitations and their association with the normal morphology to avoid misdiagnosis. Herein, we critically review the CKs expression patterns in ECs in relation to clinicopathological features and patients' outcome. We also briefly discussed the recent advantage of CKs immunohistochemical staining in the detection of EC micrometastasis.

## Introduction

**Figure 1:**
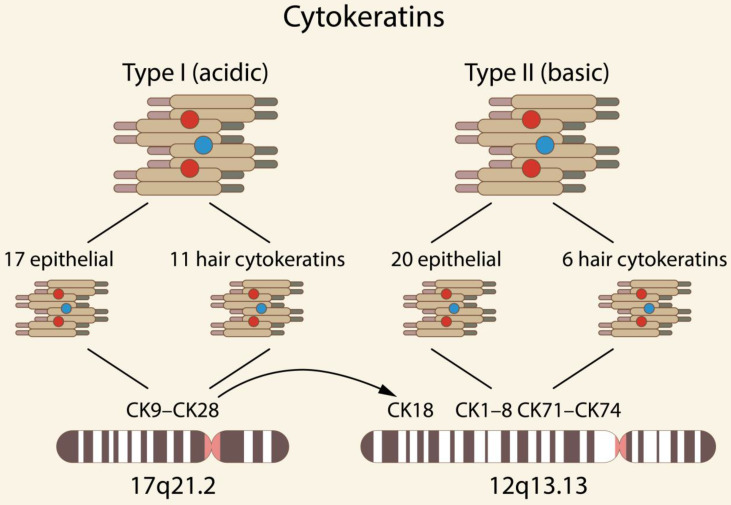
Chromosomal localization of selected types of human CKs 3.

Cytokeratins (CKs) are the largest subgroup of intermediate filament (IF) proteins, preferentially expressed in epithelial tissues, coded by keratin genes 1-2. Based on the 2D gel migrations, molecular weight and isoelectric point, they are subdivided into type I, i.e., acidic (CK9-CK28) and type II, i.e., basic (CK1-CK8 and CK71-CK74) 1, 3-4. Type I comprises 17 epithelial and 11 hair keratins, and type II comprises 20 epithelial and 6 hair keratins. Genome analyses have demonstrated that humans possess a total of 54 functional *CK* genes, i.e., 28 type I and 26 type II CKs, forming two clusters of 27 genes each on chromosomes 17q21.2 and 12q13.13 (the gene for the type I CK18 is located in the type II *CK* gene domain) 3 [Fig. 1].

**Figure 2:**
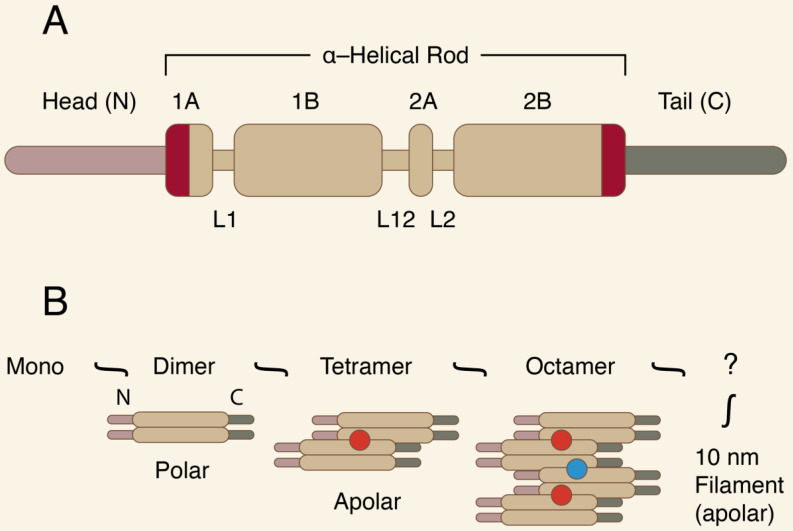
** A-B.** The IF protein organization.

CKs are resistant to degradation, show great fidelity of expression, and are very antigenic. All CKs share the common basic molecular structure of cytoplasmic IF proteins. They have a highly conserved central coil α-helical “rod” domain, which is crucial for proper filament assembly. The central domain is surrounded by non-α-helical, N-terminal “head” and C-terminal “tail” domains of various lengths 5-7. Figure 2 presents the IF protein organization.

The “rod” domain's sequence homology is usually mutual for the entire protein family, while individual features of particular CK proteins are ensured by variations in the “head” and “tail” domains. CKs are obligatory hetero-polymers, and for proper filament formation, at least one type I and one type II CK must be expressed together. Their expression is differentiation-dependent and developmentally regulated, and they are specific for different types of human epithelia. While liver epithelial cells express only one pair of CKs (CK8 and CK18), all other types of epithelia produce approximately 4 to 8 CKs 7. For example, stratified squamous epithelia express mostly CKs 1 to 6 and 9 to 17, while CKs 7, 8, 18, 19, and 20 are identified in simple squamous epithelia. Of the latter, CKs 8, 18, and 19 are the most abundant in human malignancies 8.

The point mutations in human *CK* genes (in the “rod”domain) are associated with different epithelial disorders in multiple tissue types 4, 9. Some inherited dermal human diseases exhibit cytolysis of epithelial cells, resulting in blistering of the corresponding epithelial sheets 8-10. These observations, along with several studies conducted on *CK* knockout mice and mice carrying dominant *CK* mutations, suggest that CK filaments provide mechanical support to tissue architecture and are critical for the maintenance of cell viability 9-11. In addition to their cyto-protective function, they form complex signaling platforms and interact with various proteins, such as kinases, adaptors and receptors 12-14. Moreover, they regulate different cellular processes, including protein synthesis, cell growth and differentiation 15-18.

CKs play a critical role in determining epithelial structural integrity under stressful conditions in addition to their fundamental functions in cellular proliferation, apoptosis, migration, adherence and molecular signaling 13, 18. They have been conventionally applied as a diagnostic tool in cancer 17-21. Recently, growing number of evidence suggests their importance, not only in diagnosis, but also in the regulation of the formation of epithelial tumors, as well as in the evaluation of the response to treatment and prognosis 22-27. In the literature, there are reports investigating the expression patterns of various CKs in diagnosis and therapy of epithelial ovarian cancer 28-33, and in CIN/invasive cervical cancer 34-40. It is highly debatable if assessment of CK expression pattern may serve as a potential tool for EC development and progression.

There is evidence about an active keratin role in cancer cell invasion and metastasis, highlighting their ability to transform cell shape and migration pattern through interactions with the extracellular environment 41. Specifically, co-expression of CK8/CK18 in particular type of cells (i.e., fibroblasts) with vimentin in mouse models, as well as in human melanoma and breast cancers cells *in vitro* indicates enhanced deformability and invasiveness. Moreover, it appears that sphingosylphosphorylcholine, which is abundant in the blood and ascites of ovarian cancer patients, initiates CK8 and CK18 phosphorylation, inducing reorganization in the keratin network. This contributes to changes in the cell shape and better cell migration and permeation abilities. Especially keratin phosphorylation significance was noticed in colorectal cancer progression. As shown in hepatocellular and breast carcinoma cells, CK8 on the cell surface mediates plasmin production via urokinase-type plasminogen activator pathway, which in turn augments malignant cell potential to adhere to fibronectin in the extracellular matrix and promotes detachment from primary tumor 21, 27.

So far, EC has been classically sub-divided into two major categories, endometrioid and non-endometrioid, with different clinico-pathological and molecular features 42-44. Differences between various EC histotypes have been analyzed based on profound IHC characterization with molecular genetic analysis as well 45-48.

However, recent molecular and histopathological findings recommended a more complex scenario, and new predictive tissue markers are needed to assess the risk stratification. In this context, a major finding has changed the landscape of how we approach EC today, namely, the molecular classification accomplished by the Cancer Genome Atlas (TCGA) in 2013, which subdivides EC in four distinctive subgroups 49-51. The recent ESGO/ESTRO/ESP guidelines for the management of patients with EC incorporate the molecular classification into the definition of risk groups 52.

Unfortunately, the expression pattern of CKs was not provided as a real validated value in the classification of ECs, but still microscopic evaluation with immunohistochemical staining for CK in lymph node micrometastasis, to identify small clusters of cancer cells, is a “gold” standard 53-54. In this procedure, they serve as prognostic or predictive biomarkers - even in patients with low-risk or intermediate-risk disease (except for cases showing the lack of myometrial infiltration). There is no review of CKs in EC up to now, suggesting the topic is innovative and of worth interest.

The relevant literature reviewed for this article was retrieved by searching for the terms “endometrial carcinoma”, “cytokeratins”, “micrometastasis” from 1983 to December 2021 in PubMed^®^ database. Further references were identified by analyzing the retrieved publications as well as by the authors' personal knowledge.

### Detection of CKs

CK profiling is especially valuable for poorly-differentiated carcinomas, carcinomas spreading over several organs, and, in particular, for distant metastases of an unknown primary origin 55-57.

CKs have also been recognized as prognostic indicators in a variety of epithelial malignancies. Immunohistochemical detection of CKs has become a widely established tool in clinical tumor pathology, where particularly CK5-CK8 and CK18-CK20 are routinely used 1, 31, 54.

Over the past 3 decades, a considerable amount of monoclonal antibodies, that can successfully be implemented on routinely processed tissue specimens, have been developed and are currently commercially available. These antibodies could be broadly categorized into 2 major groups. The term “broad-spectrum” often refers to the first group, which comprises antibodies that react with several CKs and usually stains nearly all types of epithelia and their derived tumors. Those individual clones or mixtures of clones (“cocktails”) in the aforementioned group are commonly used as screening reagents for the demonstration of epithelial differentiation, aiding in distinguishing a poorly differentiated carcinoma from a melanoma, lymphoma or sarcoma 52, 54. The second group includes antibodies, which recognize only a single CK peptide and whose, therefore, have a more limited immuno-reactivity 54.

AE1/AE3 is an example of a broad-spectrum CK antibody cocktail, and is probably most commonly used in pathological assessment. It is composed of the mouse monoclonal antibody AE1 that recognizes the acidic (type I) CKs 10, 14, 15, 16, and 19, and AE3 that reacts with the basic (type II) CKs 1, 2, 3, 4, 5, 6, 7, and 8. Another broad-spectrum CK antibody cocktail is MNF116. It reacts with CKs 5, 6, 8, 17, and probably 19. Similar to AE1/AE3, this antibody has the drawback of not reacting with CK18.

A brief summary of the main anti-CK antibodies used in the experimental setting is presented in Table 1.

### Expression profiles of selected CKs in human endometrial carcinomas (ECs)

Endometrioid-type carcinoma is almost always positive for CK7, CK8, CK18, and CK19 56-57, and the vast majority of the cases are CK7 positive and CK20 negative 58-62. Recently, Miyamoto *et al.* 63 reported that sero-mucinous component of EC had positive reactivity for CK7, and negative reactivity for CK20, and could be a histologic predictor for prognosis. Several studies have reported different CKs expression patterns in primary human ECs.

In one study, normal endometrial glands were usually CK19-positive 64. There was a more intense staining in the functional layer, while the basal zone epithelium generally showed weak or even focal immunoreactivity. The proliferative epithelium particularly showed a pattern of more intense staining in the basal and apical cytoplasmic segments. However, it was noticed, that only sizeable neoplastic glands in the middle part of the tumor stained strongly, while peripheral glands were characterized by weak-to-absent CKs' reactivity. In contrast, endometrioid carcinoma with areas of microcystic, elongated and fragmented glands (MELF-type) was homogeneously and strongly CK19 positive, even when the closest ''conventional''- type tumor glands revealed no reactivity 65. In a previous study of the afomentioned researchers, endometrioid-type ECs with a MELF-pattern of myometrial infiltration tended to show higher expression of CK7 and CK19 64.

In another report 66, normal endometrial glandular tissues immunostained completely with CK8 and CK18. The aforementioned CKs were mainly distributed around the nuclei, and there was no staining on the surface of the glandular epithelia or within the nucleus. In terms of EC samples, total CK8 scores ranged from 0 up to 6. At least weak and focal staining was reported in all samples, except one. CK8 was distributed mainly around the nucleus in strongly stained tissues. Basal or basal-apical staining in the cytoplasm was seen in weak or moderately stained neoplastic cells. In comparison, CK18 staining was generally more intense, with total CK18 scores ranging from 3 up to 6. Strong and complete cytoplasmic CK18 staining around the nucleus was observed in 61% of cases. Most samples showed evenly distributed cytoplasmic staining, while there was no reaction on the surface of the tumor cells or in the nucleus. Both CK8 and CK18 revealed LVSI in 82% of cases, whereas CK18 not only stained the neoplastic cells emboli and apoptotic cellular remnants in the vessels, but also the vessels themselves. CK18 also strongly stained micrometastasis of pelvic lymph nodes 66.

Interestingly, out of 10 ECs, six (60%) were positive for CK5/6 in a report of Baghla *et al.* 67. Four of these cases - pure ECs - displayed only weak positivity for CK5/6. Comparatively, the poorly-differentiated neoplasms displayed increased CK5/6 immunostaining. Other researchers demonstrated that CK5/6 expression was focal, weak or negative, which correlates completely with the data published previously 58, 68. Two out of six positive cases revealed squamous metaplasia, showing enhanced CK5/6 expression. Similarly, other authors noticed CK5/6 expression in cases of EC with squamous metaplasia 68.

In another report, Stefansson *et al.* 69 aimed at investigating the association between CK5/6 expression and specific EC phenotypes. CK5/6 expression was found in slightly less than a half of the cases. Endometrioid-type ECs with squamous differentiation (previously deemed “adenosquamous”) revealed a considerably more intense staining with CK5/6 than the pure endometrioid or serous/clear cell carcinomas. Expression of CK5/6 was mainly found in areas with squamous differentiation, but was also reported in non-squamous areas. Cases of normal endometrium, simple endometrial hyperplasia, and complex endometrial hyperplasia were also examined. In normal endometrium, staining of CK 5/6 was generally weak and focal. In comparison, nearly 30% of the dilated glands in simple endometrial hyperplasia revealed a strong cytoplasmic homogeneously distributed CK5/6 positivity. More intense positive staining was detected in areas of squamous metaplasia. In complex endometrial hyperplasia, CK5/6 expression was generally negative, apart from areas with squamous differentiation 69.

Undifferentiated carcinoma of the endometrium (UCAe) is an aggressive, under-recognized, high-grade tumor that occur either in its typical form or in conjunction with low-grade endometrioid carcinoma (i.e. dedifferentiated EC) 70. UCAe is a high-grade carcinoma that must be distinguished from endometrioid-type EC, FIGO grades 2 and 3, because of its aggressive behavior. In addition, there are immunophenotypic differences between UCAe and other types of ECs that pose problems to the pathologist while evaluating biopsies of recurrent or metastatic disease. There is a considerable morphological overlap between the typical solid growth pattern of UCAe, the solid component of EC and sarcoma, leading to frequently inaccurate pathological assessment. In the literature, the role of a selected group of immunomarkers in the distinction of UCAe from other ECs has been evaluated 71. Cases of UCAe were stained with antibodies against CK cocktail (AE1/AE3, CAM5.2, MNF116), CK8/18, CK5/6. Of these, 77% were positive for CK cocktail and CK8/18, whereas only 11% were CK 5/6 positive. Moreover, 10 out of 35 (33%) cases were diffusely positive for CK cocktail, 9 (25%) showed patchy staining, 8 (26%) showed focal staining, while 8 (26%) were negative. In addition, 14 out of 34 (41%) cases were diffusely positive for CK8/18, 7 (21%) showed patchy staining, 6 (17%) showed focal staining, and 7 (21%) were negative. CK5/6 was negative in 14 (78%) cases, 3 (17%) showed patchy staining, and only one (5%) was focally positive. The endometrioid component of all the dedifferentiated tumors was diffusely and strongly positive for all the aforementioned CK markers 71.

The use of keratin cocktail is not reliable in distinguishing UCAe from the solid component of endometrioid-type EC. Although most cases of UCAe tended to be just focally positive for CK cocktail (approximately 5%-10%), a new data published from the same Institute revealed that 54% of all cases have either patchy or diffuse expression 70. This difference might be related to the antibody dilution and retrieval methods applied. In addition, some investigators reported that CK18 may be a new epithelial marker of choice in UCAe 72. However, another study found that there was no distinct difference between the expression of CK cocktail and CK8/18 - 60% of their cases expressed the latter 71. Therefore, pankeratin and CK8/18 are equally useful in establishing the epithelial component of UCAe, especially when lymphoma, melanoma or sarcoma is considered in the expanded differential diagnosis 71.

A recently published report on two cases of dedifferentiated EC showed that the undifferentiated component was only focally positive for cytokeratin staining, while the glandular component was diffusely positive 73.

The choice of the appropriate therapeutic plans for uterine endometrioid-type EC depends on the primer and proper diagnosis of the tumor's site of origin, distinguishing primary endocervical adenocarcinomas from uterine neoplasm 42, 74-75. However, adenocarcinomas of the uterine cervix displayed a considerable overlap with EC in terms of resembling morphological features, making a precise pathological diagnosis challenging. The objective of the reported research was to compare the immunoprofiles of primary cervical adenocarcinoma and EC, using an extended panel of antibodies 76. Obtained tissue samples were immunostained with pancytokeratin, CK5/6, CK7, CK8/18, CK19, CK20, CK22, and other commercially available antibodies. Only CK8/18 revealed a remarkably higher frequency of positivity in endometrioid-type ECs relative to cervical adenocarcinomas (*p*=0.002). There were positive 66% of cervical adenocarcinoma *in situ* cases and 63% of invasive cervical adenocarcinoma cases in comparison to 94% of ECs and 91% of serous carcinoma of the uterus 76.

Five commonly-used IHC markers, including CK7, CK2 and CK34βE12, were applied to analyze their potential use is distinguishing between primary endocervical and endometrial adenocarcinomas 77. However, IHC expression pattern of antibodies did not differ in frequency from both the two primary adenocarcinomas originated from the female genital tract 77.

The following study aimed to make clear whether the immunohistochemistry of the CK 8/18 monoclonal antibody, instead of CAM 5.2, has potential use in distinguishing between endocervical adenocarcinomas and ECs 78. The IHC expression of all 3 markers, CK8, 18, and 8/18, revealed no statistically significant (*p* > 0.05) differences between the immunostaining results (positive *vs.* negative) in tumors of both gynecologic malignancies. Although CAM 5.2, which was previously thought to react with both CK8 and CK18, has been reported to be helpful in distinguishing between primary endocervical adenocarcinomas and ECs 76, the above mentioned researchers could not confirm this observation (using the true CK8/18 monoclonal antibody). Finally, CAM 5.2 was mistakenly thought to react with both CK 8 and 18, as the latest revised data suggests that CAM 5.2 reacts with CK7 and CK18, rather than CK18. The results of this study support the idea that there is a misleading impression that CK8/18 is differentially expressed in the two aforementioned gynecologic neoplasms 78.

In a study of Chinese researchers 79, the expression of CK5 decreased significantly with malignant transformation of endometrial glands (*p* < 0.05). Expression of CK5 decreased when clinical stage, histologic grading, and MI increased. These Authors finally concluded that “…malignant transformation might be accompanied by a loss of CK5 expression…” 79.

Until now, knowledge about CKs expression in endometrial carcinoma is limited; hence their implementation in clinical practice faces a considerable challenge. To sum up, based on the evidence currently available, it appears that normal endometrial glands usually stain with CK19 (especially in the functional layer), with CK8 and CK18 (mainly around the nucleus). CK19 together with CK7 are also expressed in ECs with a MELF-pattern of myometrial infiltration. It appears, that CK8 and CK18 could serve not only as potential markers for detection of EC, but also reveal its invasiveness - LSVI and micrometastasis of pelvic lymph nodes. CK8/18 is statistically significantly more frequently expressed in endometrioid-type EC compared with cervical adenocarcinoma, making the challenging diagnostics of primary tumor's site of origin easier, although some authors showed no difference in expression of CK8/18 in these two cancers. In addition, CK8/18 is as useful as pankeratin in staining the epithelial component of UCAe. Loss of CK5/6 expression is frequent in ECs and is associated with aggressive tumor behavior (poorly-differentiated neoplasms) and decreased patients' survival as well. Endometrial hyperplasia CK5/6-negative may be more suspicious to EC progression. CK5/6 also stains more intensely in ECs with squamous metaplasia.

Apart from CKs discussed earlier, it appears that EC usually stains for CK7, while expression of CK20 is usually absent 56, 62, 75, 76, 77. Interestingly, in one study, all EC specimens stained with CK22 76. In terms of broad spectrum CK antibody cocktails, there is an evidence that pankeratin is more specific for EC compared with other broad-spectrum, anti-keratin monoclonal antibody CK34ᵝE12 (CK1/5/10/14) (94% and 38.1%, respectively) 76,77.

**Figure 3:**
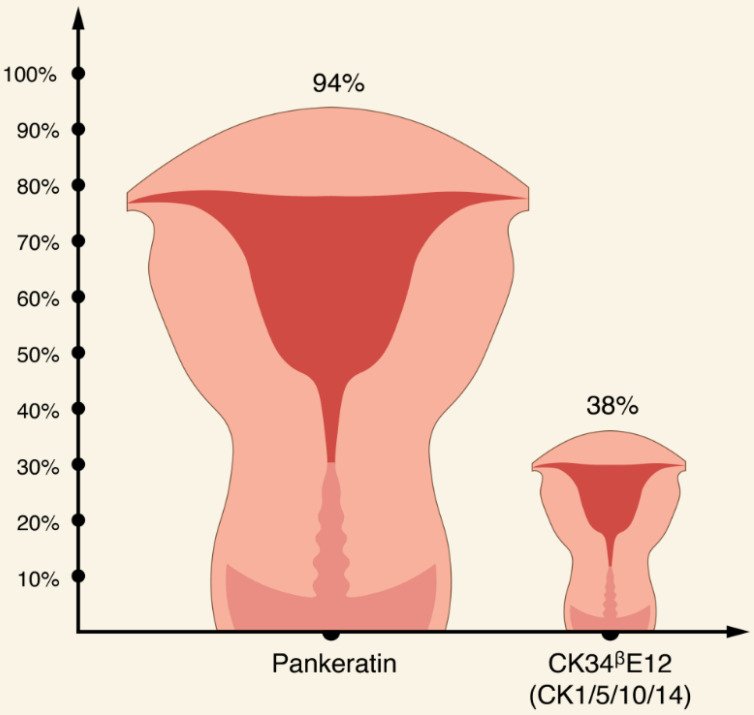
Comparison of the expression patterns of pankeratin and CK1/5/10/14 in ECs 76-77.

**Figure 4:**
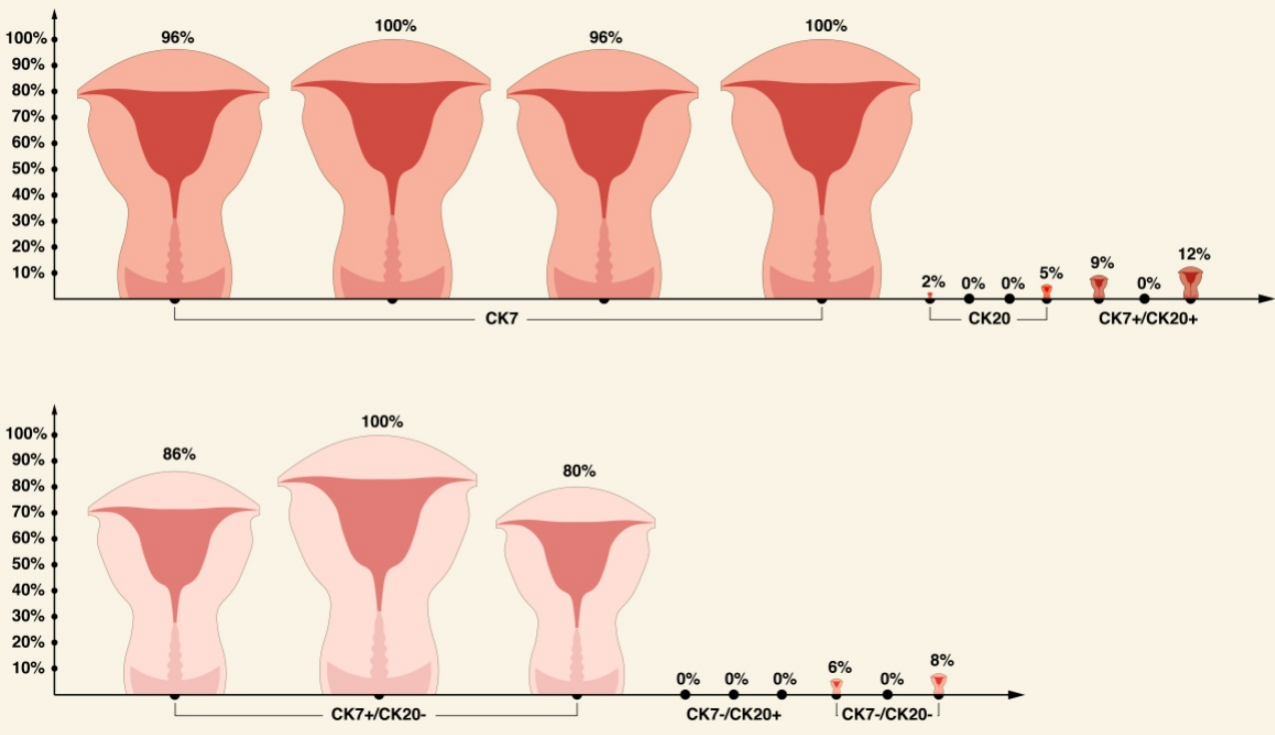
Comparison of CK7 and CK20 expression in EC based on literature review 56, 62, 75-77.

**Figure 5:**
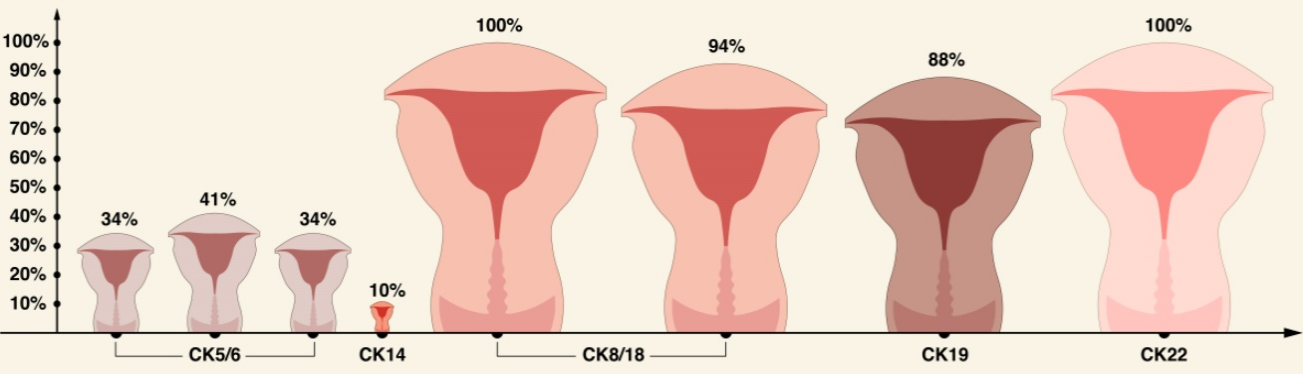
Comparison of CK5/6, CK14, CK8/18, CK19, and CK22 in ECs based on literature review 56, 69, 76.

A brief summary of CKs expression pattern in ECs is shown in Table 2 and in Figures 3-5.

### Prognostic significance and relation with clinicopathological features of CK expression patterns in primary human ECs

It is highly debatable, if assessment of CK expression could serve as a potential tool for establishment of patients' outcome. One study showed that there were no significant differences between the favorable and unfavorable outcome of the early-stage EC compared to CK7 and AE1/AE immunoreactivity 80.

At a practical level, the fact that MELF-type invasion ECs stained strongly with CK19 could encourage the use of this staining as an additional immunohistochemical marker, which may be helpful in showing the extent of myometrial spread, including the unremarkable attenuated glands and single infiltrating cells that often extend beyond the promptly evident conventional tumor areas 81. Additionally, as CK19 staining also accentuates intravascular neoplastic cells, foci of LVSI could be identified more accurately. Often subtle nodal metastases occurring in MELF-type ECs may also be precisely recognized with CK19 immunostaining 80, 82. Recently, Rabe *et al.* 83 suggested the routine CKs staining on sentinel lymph nodes in MELF-pattern ECs to detect metastases and isolated tumor cells.

Similarly, another study demonstrated that staining with CK8 and CK18 may be useful in detecting LVSI, emphasizing that higher stages of ECs had statistically higher LVSI (*p* ≤ 0.005) 66. Although EC samples stained variably with CK8 and CK18, there were no statistically significant differences between tumor grade and total CK8 and CK18 staining scores (*p* = 0.187 and *p* = 0.675, respectively). Comparably, the total CK8 and CK18 staining scores did not correlate significantly with the clinical stage of the disease (*p* = 0.412 and *p* = 0.129, respectively) 66.

As aforementioned, the presence of CK5/6 immunostaining was more frequent in endometrioid-type tumors with squamous differentiation 69. The loss of CK5/6 expression in ECs (observed in slightly more than a half of the cases) was significantly associated with a higher FIGO stage, increased tumor cell proliferation assessed by Ki-67 expression, reduced *β*-catenin expression, MI as well as an unfavorable patient outcome (66% 5-year survival *vs.* 86% 5-year survival (preserved CK5/6 expression), *p* = 0.0001). For patients with the endometrioid-type EC subgroup, the corresponding figures were 71% and 89%, respectively (*p = 0.0004*). Comparably, patients with localized disease (characterized by FIGO stages I/II) and with the lack of CK5/6 expression showed significantly reduced survival (79% at 5 years) compared to patients with preserved expression (94%; *p =* 0.001). CK5/6 turned out to have an independent prognostic value in a multivariate model, including other well-known prognostic factors (histologic type, histologic grade, vascular invasion, myometrial invasion and clinical stage). By substituting histologic grade with nuclear grade, CK5/6 still had an independent prognostic impact (HR = 2.0; *p = 0.02*), in addition to the histologic type (HR = 2.7; *p = 0.013*), vascular invasion (HR = 2.9; *p = 0.003*), myometrial infiltration (HR = 2.6; *p = 0.005*) and the clinical stage (HR = 5.3; *p < 0.0001*). When cases of localized disease were studied separately, the variables: histologic type (HR = 3.9; *p = 0.012*), vascular invasion (HR = 17.3; *p < 0.0001*) and CK5/6 (HR = 3.9; *p = 0.002*), still had an independent prognostic impact. Interestingly, in a separate multivariate analysis of the endometrioid subgroup, the role of CK5/6 expression appeared to be of borderline significance (HR = 2.0; *p = 0.09*), while histologic grade (HR = 2.3; *p = 0.03*), vascular invasion (HR = 4.4; *p = 0.001*) and clinical stage (HR=6.1; *p < 0.0001*) obtained still an independent prognostic value. As a conclusion, that loss of CK5/6 expression is frequent in endometrioid-type ECs and is associated with aggressive tumor behavior and decreased patients' survival rates. In addition, it is an adverse prognostic marker in multivariate analysis 69.

In another report, the investigators assessed the value of the molecular biomarkers in endometrial hyperplasia progressed to uterine carcinoma 84. In total, 142 cases of endometrial hyperplasia were available for analysis (134 non-endometrial intraepithelial neoplasia; 8 EIN). The median follow-up was 57 months (range, 12-283 months). Interestingly, CK5/6-positive endometrial hyperplasia progress to EC only in 1.2%, whereas the corresponding figure for CK5/6-negative cases was 13.8% (*p* = 0.001, HR 13.6) 84.

It is worth citing the results of the study by Bai *et al.* 85 who investigated CK17 IHC pattern in a cohort of 117 high-grade ECs. CK17 immunostaining correlated with decreased OS (HR, 1.8, *p* = 0.0488). Finally, they suggest that CK17 IHC test results could be clinically applied to make the final decision related to therapeutic intervention 85.

### Cytokeratins and EC micrometastases

The presence of micrometastases in regional lymph nodes is one of the most core risk factor determining the widespread of EC corresponding with patients' outcome 86-88. Novel insights in molecular biology have led to the development of more sensitive and sophisticated methods for detecting micrometastasis to lymph nodes in female genital tract carcinomas, including ECs. CKs staining of lymph nodes from EC patients has been proven to be more sensitive than traditional histopathologic evaluation for the detection of micrometastasis. In a study by Bosquet *et al.* 89 CK staining was performed with AE1/AE3 antibodies. All lymph nodes proved to have (micro)metastasis by examination of frozen sections during the initial surgery were also shown to be positive during secondary assessment with hematoxylin and eosin-staining. However, 12.5% of all lymph node-negative cases had micrometastasis revealed by CK staining. One of these patients developed recurrent disease in the para-aortic lymph nodes and, unfortunately, died from the disease after almost 3 years of follow-up. This study clearly highlights the clinical significance of micrometastasis investigation. Moreover, an approach incorporating CK staining during lymph nodes assessment could improve the risk evaluation, especially in the highly-risk EC women 89.

In another report, investigators aimed to evaluate the clinic-pathological significance of CKs expression staining in lymph nodes with unconfirmed metastasis, which were assumed to be responsible for the recurrent disease of patients treated for early-stage EC 90. They examined retrospectively 304 pelvic lymph nodes and 46 primary tumors resected from 46 patients with ECs. The study sample was comprised of 36 women with stage I disease and 10 patients with stage IIIC disease. In the subgroup of stage IIIc, pancytokeratin expression was detected in all 13 lymph nodes with metastasis and also in 20 out of 66 (30.3%) lymph nodes without previously detected metastasis. Furthermore, CK expression was demonstrated in 37 out of 225 (16.4%) lymph nodes with unconfirmed metastasis in the subgroup of stage I disease. In the latter subgroup, CK expression in lymph nodes was detected in 10 out of 14 (71.4%) patients with LVSI. This last was remarkably more frequent than the expression in 4 out of 22 (18.2%) patients without LVSI. More than a third of patients with lymph nodes expressing CK also had a recurrent tumor within the pelvic cavity, whereas all patients with CK-negative lymph nodes showed no recurrence of disease within 5 years after the primary surgery. A relationship between CK expression in the lymph nodes and LVSI of the primary tumor was detected, but there was no association between CK expression and the histologic grade or depth of myometrial invasion. In the multivariate analysis of the subgroup of stage I disease, CK-positive lymph nodes were identified as an independent risk factor for disease recurrence 90.

It was suggested that the detection of CK20 could substitute traditional histopathologic methods in the diagnosis of micrometastasis in lymph nodes of EC patients 91. The presence of metastases in 10% of all patients was demonstrated by histopathologic examination. These patients were also CK20-positive. Of the remaining 90% patients with negative histopathologic results, 33% were CK20-positive. Results of the meta-analysis also suggest that CK 20 is more sensitive than traditional histopathologic method with H&E (sensitivity was 94.5 and 91%, respectively) 92. These results encourage the consideration of the use of CK20 more frequently in order to precisely detect micrometastasis in lymph nodes of patients with EC and take the appropriate treatment steps in order to prevent disease recurrence 91.

Altogether, the immunohistochemical staining of CKs in lymph nodes with undetected metastasis by conventional methods could serve as a predictor of occult metastasis to these nodes and increases the likelihood of disease recurrence in early-stage EC 90-93.

There are manuscripts describing advanced molecular techniques (for example qRT-PCR) in detecting EC lymph node micrometastases 94-95. These techniques are even more sensitive than IHC, but they also carry high false-positive results 94-95. Interestingly, Pappa *et al.* 94 assumed that “..qRT-PCR exhibits a better diagnostic accuracy compared with IHC (for the detection of lymph node micrometastasis in cervical, endometrial and vulvar cancer), while CK19 displays a consistent pattern of detection compared to carbonic anhydrase 9…”.

Moreover, OSNA (One-Step Nucleic Acid Amplification) method, based on CK19 mRNA concentration for the detection of lymph-node metastases in EC, has been reported 96-98. OSNA showed high sensitivity (87.5%-100%) and specificity (82%-100%), suggesting an efficient intraoperative tool for the molecular detection of LNM, especially in early-stage ECs 97. Additionally, it may also serve as a useful alternative to conventional pathological diagnosis of EC lymph node metastasis. Finally, Diestro *et al.* 98 reported that “…OSNA has recently received EC marking for the detection of lymph node metastases in endometrial and cervical cancer, allowing its use in routine practice”.

There is one retrospective study from Germany, analyzing follow-up of 428 patients (302 patients with node-negative EC without adjuvant treatment, 95 with nodal micrometastases who received adjuvant treatment and 31 with nodal micrometastases who did not receive adjuvant treatment) 99. They showed that without adjuvant therapy, the DFS in the cohort of patients with micrometastasis was significantly reduced and adjuvant therapy was associated with improved DFS comparable to the DFS of node-negative patients 99.

Despite the fact of introducing the molecular classification of EC accomplished by the Cancer Genome Atlas (TCGA) in 2013, the NCCN guidelines still recommend mapping of lymph nodes with ultrastaging techniques in the form of H&E and pankeratin staining 100.

Recently, Xu *et al.* 101 published an interesting study in which the serum CK19 value was analyzed in EC patients undergoing surgical intervention. Interestingly, CK19 level could predict the possibly risk of ovarian metastases, and therefore “…the necessity of incorporating serum CK19 measurement into the pre-operative evaluation of EC, especially as extension of current standard approach with ovarian preservation counselling” is advocated 101.

## Conclusions

CKs play a major role in determining epithelial structural integrity under stressful conditions in addition to their fundamental functions in cellular proliferation, apoptosis, migration, adherence, and molecular signaling. They have been conventionally used as a diagnostic tool in cancer. Recently growing evidence suggests their importance not only in diagnosis but also in the regulation of the formation of epithelial tumors as well as in the evaluation of the response to treatment and the prognosis. A significant correlation is exhibited by the expression of particular CK types and tumor phenotypes, aiding in distinguishing between aggressive types of carcinoma, the specific localization of the tumor, the chance of invasion and metastasis, as well as the likelihood of recurrence. In terms of EC, CK5/6, CK7, CK8, CK17, CK18, CK19 appear to be the most promising diagnostic and prognostic markers. CK staining of lymph nodes from EC patients has been proven to be more sensitive than traditional histopathologic evaluation for the detection of micrometastasis. The immunohistochemical staining of selected CKs in lymph nodes with undetected micrometastasis by conventional methods could serve as a predictor of occult micrometastasis increasing the likelihood of disease recurrence. However, the underlying pathology diagnostic methods in gynecological tumors still depend on the histologic evaluation of the hematoxylin and eosin-stained slides. Immunohistochemical stains should be evaluated with a proper comprehension of their limitations (i.e. cross-reactivity) and their association with the normal morphology to avoid misdiagnosis. Extensive investigation into the multifunctional role of CKs in malignant tumors will probably result in the unfolding of upgraded diagnostic and prognostic markers with more potent therapeutic implications in EC.

## Figures and Tables

**Table 1:** Basic information about the main anti-CK antibodies used in the experimental setting 53.

Clone	Reactivity	Immunogen	Remarks	Expression in cancers
AE1/AE3	CK1-6, CK8, CK10, CK14, CK15, CK16, CK19.	Epidermal keratin	Does not react with CK18 (some sources have added clone 5D3, which reacts with CK18 and CK8). Cross-reacts with glial fibrillary acidic protein.	Negative in hepatocellular, adrenal cortical, some renal cell carcinomas, renal oncocytomas.
KL1	CK1, CK2, CK5-8, CK11, CK14, CK16-CK18.	Human keratin isolated from epidermal stratum corneum	One of the most sensitive anti-keratin antibodies. Cross-reacts with normal brain tissue and astrocytic tumors.	Stains large majority of hepatocellular carcinomas and renal epithelial tumors.
OSCAR	CK7, CK8, CK18, CK19.	Keratin extract from RT-4 and MCF-7 cell lines	Does not react with with normal brain tissue or gliomas.	Limited evidence.
MAK-6 (KA4 & UCD/PR10.11)	CK8, CK14, CK15, CK16, CK18, CK19.	KA4 - human sole epidermis UCD/PR10.11 - antigen purified from MCF-7 tissue culture media	Cross-reactivity with neural tissue.	Limited evidence. Indications exist, that this coctail stains all squamous cell carcinomas, majority of adenocarcinomas, transitional cell carcinomas, carcinoid tumors, undifferentiated carcinomas.
34βE12	CK1, CK5, CK10, CK14.	Human stratum corneum	Sometimes erroneously referred to as keratin 903.	Stains squamous cell carcinomas, nasopharyngeal carcinomas, thymomas. Usually does not stain prostatic and colorectal adenocarcinomas, follicular thyroid and hepatocellular carcinomas.
CAM 5.2	CK7, CK8, CK18, CK19.	Human colorectal carcinoma cell line HT24	Reactivity restricted primarily to CK8- screening marker for epithelial differentiation. May stain astrocytic tumors.	Reacts with hepatocellular carcinomas, lung and colorectal adenocarcinomas, ovary serous and endometrioid carcinomas, lobular breast carcinoma. Does not stain adrenal cortical carcinomas.

**Table 2:** CK expression pattern in primary human ECs based on literature review.

CKs	Total	Positive	n(%)	Reference
CK7	55	53	96	56
	10	10	100	62
	53	51	96	76
	21	21	100	77
CK20	95	2	2	56
	10	0	0	62
	52	0	0	76
	21	1	4.8	77
CK7+/CK20+	35	3	9	56
	10	0	0	62
	25	3	12	75
CK7+/CK20-	35	30	86	56
	10	10	100	62
	25	20	80	75
CK7-/CK20+	35	0	0	56
	10	0	0	62
	25	0	0	75
CK7-/CK20-	35	2	6	56
	10	0	0	62
	25	2	8	75
CK5/6	27	10	34	56
	19	81	41	69
	53	18	34	76
CK14	10	1	10	56
CK8/18	20	20	100	56
	51	48	94	76
CK19	48	42	88	76
CK22	49	49	100	76
Pankeratin	50	47	94	76
CK34ᵝE12 (CK1/5/10/14)	21	8	38.1	77

## Abbreviations

CIN: Cervical Intraepithelial Neoplasia
CK: Cytokeratin
DFS: Disease Free Survival
EC: Endometrial Cancer
EIN: Endometrial intraepithelial neoplasia
ESGO: European Society of Gynaecological Oncology
ESP: European Society of Pathology
ESTRO: European Society of Radiation Oncology
FIGO: Federation Internationale de Gynaecologie et d'Obstetrique
HPV: Human Papillomavirus
IHC: Immunohistochemistry
IF: Intermediate Filament
LNM: Lymph Node Metastasis
LVSI: Lymphovascular Space Invasion
MELF: Microcystic, Elongated and Fragmented
MI: Myometrial Invasion
NCCN: National Comprehensive Cancer Newtork
OSNA: One-Step Nucleic Acid Amplification
OS: Overall Survival
UCAe: Undifferentiated Carcinoma of the Endometrium
qRT-PCR: Quantitative Real Time-PCR
